# High-resolution mapping of genes involved in plant stage-specific partial resistance of barley to leaf rust

**DOI:** 10.1007/s11032-017-0624-x

**Published:** 2017-03-16

**Authors:** F. K. S. Yeo, R. Bouchon, R. Kuijken, A. Loriaux, C. Boyd, R. E. Niks, T. C. Marcel

**Affiliations:** 1 Plant Breeding, Wageningen University and Research, Droevendaalsesteeg 1, 6708PB, 6700 AJ Wageningen, the Netherlands; 2 Department of Plant Science and Environmental Ecology, Faculty of Resource Science and Technology, University Malaysia Sarawak, 94300 Kota Samarahan, Sarawak, Malaysia; 3 Department of Crop and Soil Sciences, Washington State University, Pullman, WA 99164-4660 USA; 4 UMR BIOGER, INRA, AgroParisTech, Université Paris-Saclay, 78850 Thiverval-Grignon, France; 5 Greenhouse Horticulture, Wageningen University and Research, 6700 AP Wageningen, the Netherlands

**Keywords:** High resolution mapping, Quantitative trait locus (QTL), *Puccinia*, Barley, Resistance

## Abstract

Partial resistance quantitative trait loci (QTLs) *Rphq11* and *rphq16* against *Puccinia hordei* isolate 1.2.1 were previously mapped in seedlings of the mapping populations Steptoe/Morex and Oregon Wolfe Barleys, respectively. In this study, QTL mapping was performed at adult plant stage for the two mapping populations challenged with the same rust isolate. The results suggest that *Rphq11* and *rphq16* are effective only at seedling stage, and not at adult plant stage. The cloning of several genes responsible for partial resistance of barley to *P. hordei* will allow elucidation of the molecular basis of this type of plant defence. A map-based cloning approach requires to fine-map the QTL in a narrow genetic window. In this study, *Rphq11* and *rphq16* were fine-mapped using an approach aiming at speeding up the development of plant material and simplifying its evaluation. The plant materials for fine-mapping were identified from early plant materials developed to produce QTL-NILs. The material was first selected to carry the targeted QTL in heterozygous condition and susceptibility alleles at other resistance QTLs in homozygous condition. This strategy took four to five generations to obtain fixed QTL recombinants (i.e., homozygous resistant at the *Rphq11* or *rphq16* QTL alleles, homozygous susceptible at the non-targeted QTL alleles). In less than 2 years, *Rphq11* was fine-mapped into a 0.2-cM genetic interval and a 1.4-cM genetic interval for *rphq16*. The strongest candidate gene for *Rphq11* is a phospholipid hydroperoxide glutathione peroxidase. Thus far, no candidate gene was identified for *rphq16*.

**Electronic supplementary material:**

The online version of this article (doi:10.1007/s11032-017-0624-x) contains supplementary material, which is available to authorized users.

## Introduction

Partial resistance of barley against barley leaf rust (*Puccinia hordei*) results in a reduced epidemic, despite a compatible infection type (Parlevliet 1979). The reduced rate of epidemic progress is due to a lower infection frequency, lower sporulation rate and longer latency period of the pathogen on barley accessions with high levels of partial resistance (Parlevliet 1979). Partial resistance is a prehaustorial resistance, where failed attempts to form haustoria are associated with cell wall reinforcements, called papillae (Niks 1986; O’Connell and Panstruga 2006). The failure of a proportion of the haustorium formation reduces the capacity for nutrient extraction from the plant and for delivery of pathogenicity-promoting effectors into the plant cells (Niks 1986; Catanzariti et al. 2007; de Jonge et al. 2011). This prehaustorial mechanism of resistance is also implicated in non-host resistance of barley to heterologous rust fungi like *Puccinia recondita* and *P. triticina* (Niks 1983, 1989).

Partial resistance of barley to *P. hordei* is polygenically inherited and is supposed to act on a minor-gene-for-minor-gene model (González et al. 2012; Marcel et al. 2008; Niks et al. 2000; Parlevliet and Zadoks 1977; Qi et al. 1999; Niks et al. 2015). There is an abundance of quantitative trait loci (QTLs) for partial resistance against barley leaf rust. To date, at least 20 partial resistance QTLs against barley leaf rust have been mapped in different bi-parental mapping populations. In each barley mapping population, a different set of QTLs was identified, with few QTLs shared among the populations. The explained phenotypic variation per QTL ranges from around 50 to 50% (Jafary et al. 2008; Marcel et al. 2008; Marcel et al. 2007b; Niks et al. 2000; Qi et al. 1999; Qi et al. 1998).

QTL mapping studies indicated that the resistance QTLs can be effective across different stages of plant development or only at specific stages (Qi et al. 1998), which was confirmed on QTLs that were introgressed into near isogenic lines (NILs) (Wang et al. 2010). Plant growth stage-dependent effects of resistance QTLs have also been observed in other plant pathosystems (Aghnoum et al. 2010; Dedryver et al. 2009; Shankar et al. 2008; Steffenson et al. 1996). Consequently, different sets of QTLs will protect barley plants against leaf rust at different growth stages. It is important to know the effect of QTLs at different growth stages before designing breeding strategies or to engage in a positional cloning procedure.

The cloning of several genes responsible for partial resistance of barley to *P. hordei* will allow elucidation of the molecular basis of this type of plant defence (Marcel et al. 2007a). Fine mapping and positional cloning require the evaluation of very large numbers of plants in a controlled environment and a similar physiological condition, which is much easier to achieve at seedling stage. To date, no QTL for resistance to rust fungi or powdery mildew has been cloned in barley. However, three large-effect resistance QTLs have been cloned in rice: two against *Magnaporthe oryzae* (Fukuoka et al. 2009; Hayashi et al. 2010) and one wide-spectrum QTL against *Rhizoctonia solani* and *M. oryzae* (Manosalva et al. 2009). There are three cloned QTLs in wheat; one against *Puccinia striiformis* (Fu et al. 2009) and two wide-spectrum QTLs against the three rust species *P*. *triticina*, *P. striiformis* and *P. graminis* and against *Blumeria graminis* (Krattinger et al. 2009; Moore et al. 2015). All the genes cloned so far belong to different gene families and are involved in different molecular functions, suggesting a wide diversity of mechanisms underlying partial resistance.

A map-based cloning approach requires to fine-map the QTL in a genetic window sufficiently narrow to make physical mapping feasible. This approach requires the effect of the QTL to be sufficiently clear to infer the QTL genotype from its phenotype; the phenotypic variation explained by the QTL should be more than 10% according to Kou and Wang (2012). Once identified in a segregating mapping population, the commonly followed strategy to fine-map QTLs requires the introgression of the QTL in a NIL. In non-isogenic plant materials, other QTLs may be segregating in the genetic background blurring the determination of the phenotypic effect of the QTL of interest. The NIL carrying the targeted QTL is crossed with its recurrent parent to “Mendelise” the QTL in the resulting progeny. Then, a selection of plants recombining at the QTL-containing chromosome region (i.e., sub-NILs) and the evaluation of their phenotype allow to pinpoint the targeted QTL into a refined genetic position (Han et al. 1999; Marcel et al. 2007a; Xue et al. 2010; Zhou et al. 2010). Fine mapping using this strategy is effective but very laborious and time consuming in generating the plant materials, marker development and genotyping.

*Rphq11* (resistance is dominant) and *rphq16* (resistance is recessive) are partial resistance QTLs against *P*. *hordei* isolate 1.2.1 that were mapped in seedlings of the mapping populations Steptoe/Morex (S/M) and Oregon Wolfe Barleys (OWB; Dom/Rec), respectively (Marcel et al. 2007b). They are effective at seedling stage in their respective mapping population, each explaining approximately 30% of the phenotypic variance. *Rphq11* was mapped at seedling stage near the middle of chromosome 2HL, and the resistance allele was contributed by Steptoe. It was also detected by Chen et al. (2010) as an expression QTL (eQTL) co-locating with the phenotypic QTL (pQTL) in the same mapping population. Six candidate genes were suggested by those authors that may explain *Rphq11*. *rphq16* was mapped at seedling stage near the telomeric region of chromosome 5HL, and the resistance allele was contributed by Dom.

The first objective of this study was to test whether *Rphq11* and *rphq16* are also effective at adult plant stage. The second objective was to fine-map *Rphq11* and *rphq16* using an approach aimed at speeding up the development of plant material and simplifying its evaluation with the final aim of cloning them. *Rphq11* and *rphq16* qualify for map-based cloning to study partial resistance because of sufficient size of phenotypic effect at seedling stage.

## Materials and Methods

### Inoculum

All the disease tests in this study were done with *P. hordei* isolate 1.2.1 (*Ph*.1.2.1), the same isolate as used by Marcel et al. (2007b). This isolate is a monospore purification of the isolate 1.2 collected in the Netherlands in 1971 (Parlevliet 1976).

### QTL mapping on adult plants

The doubled haploid (DH) mapping populations S/M (Kleinhofs et al. 1993) and OWB (Costa et al. 2001) were used to map QTLs for non-hypersensitive quantitative resistance at adult plant developmental stage, heading stage Z51–Z55 on Zadoks’ growth scale (Zadoks et al. 1974). The prehaustorial, non-hypersensitive resistance conferred by *Rphq11* and *rphq16* was confirmed by microscopic observations (data not shown). The S/M population comprises 150 DH lines, and the OWB population comprises 94 DH lines. For QTL mapping, all marker positions were extracted from the “Barley, Integrated, Marcel 2009” linkage map [linkage map and segregating marker data are available at http://wheat.pw.usda.gov/GG2/index.shtml; (Aghnoum et al. 2010)]. Data comprise 3561 segregating markers in S/M and 882 in OWB. Skeletal maps were generated for S/M and OWB by selecting markers homogeneously distributed over the integrated map, spaced at approximately 1–5-cM intervals.

The disease phenotyping of the entire populations was repeated three times—at different periods of the year. In the first and second repetitions, we evaluated three individual plants (biological replicates) per DH line. The third repetition was performed at a different glasshouse facility regulated with the same climatic conditions, and only one individual plant per DH line was evaluated. The parental lines Steptoe and Morex or Dom and Rec and the reference barley lines L94 and Vada were included in each experiment. Parental and reference lines were sown continuously every 3 days from 1 week before to 1 week after the sowing of the DH lines of the two mapping populations. For each line, three seeds were sown in a pot of 15 cm diameter and 14 cm height. For the first and second repetitions, all three plants in a pot were scored. For the third repetition, one out of three plants was selected randomly for scoring. To ensure the uniformity of the developmental stage of the plants at the time of inoculation, plants of a mapping population were divided into three to four subgroups based on their heading date. For each subgroup, plants of parental and reference lines with a similar developmental stage were added.

Plants were inoculated after the flag leaf was unfolded (around heading stage Z52). Per pot, 1 mg of spores diluted 10 times with *Lycopodium* spores was used as inoculum. Before the inoculation, the pots were lined up two by two. Then, the inoculum was dusted over the plants as uniformly as possible. The inoculated plants were then placed in a humidity chamber overnight (8 h) at 100% relative humidity in the dark at 18 °C to allow the spores to germinate. After incubation, the plants were transferred to a greenhouse compartment where the temperature was set at 20 ± 3 °C during daytime and about 16 °C at night, with 30–70% relative humidity.

The flag leaf of the three plants in each pot was scored for latency period (LP50A). It was scored daily by counting the mature pustules on a marked area of the flag leaf until all the pustules matured. Immature colonies are seen as small pale green or yellowish flecks on the leaves. Those flecks turn orange at maturation, indicating the beginning of sporulation by the colony (Niks et al. 2015). Latency period estimates the period of time in hours at which 50% of the total number of pustules are mature. It is among the most informative measures of barley partial resistance to leaf rust and is relatively easy to evaluate (Niks et al. 2011; Parlevliet 1979). The relative latency period (RLP50A) was calculated, relative (in %) to the LP50A on Steptoe for S/M and to the LP50A on Dom for OWB.

ANOVA was performed using GenStat® 14th edition (VSN International Ltd. 2011). QTLs were mapped using MapQTL®6 (van Ooijen 2009). QTL mapping was performed for each repetition independently. A permutation test with 1000 permutations was performed for each repetition to set the LOD threshold to declare a QTL. The confidence interval of a QTL is the estimated LOD-2 support interval.

The data for heading date (HD) and plant height (PH) of S/M were downloaded from GrainGenes (http://wheat.pw.usda.gov/ggpages/SxM/phenotypes.html) and described by Hayes et al. (1993). The QTLs for HD and PH were mapped on the S/M skeletal map generated from the barley integrated map [(Barley, Integrated, Marcel 2009; (Aghnoum et al. 2010)].

### Dominance of phenotypic expression for the target QTLs

Previous QTL mapping studies at seedling stage revealed in the S/M population two QTLs, *Rphq11* and *Rphq15*, for which the resistance alleles were contributed by Steptoe. In the OWB population, resistance alleles of two QTLs, *rphq16* and *Rphq17*, were contributed by Dom (Marcel et al. 2007b). Steptoe and Dom were individually crossed with the experimental line SusPtrit, and their F_1_ backcrossed to SusPtrit (Atienza et al. 2004). Molecular markers flanking the QTLs were used to select for the susceptibility QTL alleles at *Rphq15* and *Rphq17* and to select for the resistance QTL alleles at *Rphq11* and *rphq16*. *Rphq11* and *rphq16* were finally introgressed into SusPtrit by backcrossing over five generations for *Rphq11* and six generations for *rphq16* to obtain NILs (Supplementary Material, Fig. S1). Details and results obtained with the NILs will be published elsewhere.

Steptoe/SusPtrit F_2_ plants being heterozygous at *Rphq11* and homozygous susceptible at *Rphq15* were selected with three molecular markers for *Rphq11* (Bmag0125, GBM1062, GBMS244; cf. Supplementary Material Table S1) and three molecular markers for *rphq15* (scssr09398, GBM033, MWG966; Supplementary Material Table S3). Three F_2_ plants were selected from which 97 F_3_ plants were genotyped with flanking markers and phenotyped to perform a dominance study of *Rphq11* (Supplementary Material, Fig. S1)*.*

Dom/SusPtrit BC_1_ plants being heterozygous at *Rphq16* and homozygous susceptible at *Rphq17* were selected with four molecular markers for *rphq16* (ABG390, ABG391, GMS002, scssr09041; cf. Supplementary Material Table S2) and two microsatellite markers for *rphq17* (Bmac0067, Bmag0136; Supplementary Material Table S3). Two BC_1_ plants were selected from which 52 BC_1_S_1_ plants were genotyped with flanking markers and phenotyped to perform a dominance study of *rphq16* (Supplementary Material, Fig. S1)*.*

The first leaves of the 97 F_3_ seedlings for *Rphq11* and 52 BC_1_S_1_ seedlings for *rphq16* were inoculated with *Ph.*1.2.1 following the method of Qi et al. (1998). Their latency period was scored (LP50S) and the relative latency period (RLP50S) calculated, relative to SusPtrit (in %). The plants were grouped according to their QTL allele, homozygous Steptoe or Dom (AA), and heterozygous (AB) and homozygous SusPtrit (BB), to estimate the effect and the dominance or recessiveness of *Rphq11* and *rphq16*. Plants that had a recombination between the QTL flanking markers were excluded from the analysis. The data were analysed with unbalanced one-way ANOVA using GenStat® 14th edition (VSN International Ltd. 2011).

### Fine mapping *Rphq11* and *rphq16*

Among the 97 F_3_ plants for *Rphq11* and among the 52 BC_1_S_1_ plants for *rphq16*, there were 12 and 18 recombinant plants, respectively. These plants were grown to set seeds which were then used to identify plants with homozygous recombination. These plants were then homozygous recombinants at the *Rphq11* and *rphq16* QTL regions, homozygous susceptible at the *Rphq15* and *Rphq17* QTL regions, but their genomic background was still segregating. For simplicity, these plants will be called “fixed QTL recombinants” from this point onwards. These fixed QTL recombinants were used to refine the positions of *Rphq11* and *rphq16* (data not shown). Based on the refined positions, new flanking markers were selected for *Rphq11* (GBS0512 and GBMS244) and for *rphq16* (scsnp03275 and GMS002) (Supplementary Material Tables S1 and S2).

In order to further fine-map *Rphq11* and *rphq16*, the F_3_ plants heterozygous for *Rphq11* and BC_2_ plants heterozygous for *rphq16* were selfed to produce a large number of seeds. New recombinants for *Rphq11* and *rphq16* were identified by screening the F_4_ and BC_2_S_1_ plants with the new flanking markers for *Rphq11* (GBS0512 and GBMS244) and *rphq16* (scsnp03275 and GMS002). The same markers were used to identify fixed QTL recombinants for both QTLs in the subsequent generation. The fixed QTL recombinants were then genotyped with all available molecular markers located in the QTL regions to generate high-resolution genetic maps around *Rphq11* and *rphq16*.

The fixed QTL recombinants were subjected to four rounds of disease tests for *Rphq11* and three for *rphq16* (Supplementary Material, Fig. S2). At each round, a different subset of the fixed QTL recombinants was strategically selected based on previous results of disease tests in order to progressively refine the map position of the QTL. This strategy allowed more individuals to be tested per fixed QTL recombinant at each round (from 5 to 10 individuals), increasing the confidence in the phenotype. SusPtrit was included in all disease tests as susceptible reference. The order of the fixed QTL recombinants was randomised within each experimental round, but individuals from the same fixed QTL recombinant were sown next to each other.

Disease tests were performed at seedling stage following the method of Qi et al. (1998). The LP50S was measured. The relative latency period on seedlings (RLP50S) was calculated by setting SusPtrit at 100. Data from different rounds of disease test were analysed together under a linear mixed model with GenStat® 14th edition (VSN International Ltd. 2011). The significance of difference in mean RLP50S between fixed QTL recombinants and SusPtrit was determined based on the least significant difference (LSD, *P* < 0.05).

To recover rare recombinant plants, genomic DNA of the plant materials for recombinant screening was extracted following the method of Wang et al. (1993), adjusted for a 96-well format. To select fixed QTL recombinants, the sbeadex® maxi plant kit (LGC Genomics) was used to isolate DNA of recombinant plants.

### Marker saturation of *Rphq11* and *rphq16* intervals

Two approaches were followed to develop 20 molecular markers in the approximately 13-cM interval of *Rphq11* and to develop 27 molecular markers in the approximately 25-cM interval of *rphq16*. All the markers developed were polymorphic in SusPtrit/Steptoe and SusPtrit/Dom, respectively.

#### Approach I

Molecular markers that mapped within the intervals of *Rphq11* and *rphq16* on the integrated map (GrainGenes: Barley, Integrated, Marcel 2009) were targeted for generating new PCR-based markers segregating in our material. Sequence information of targeted restriction fragment length polymorphism (RFLP) markers and transcript-derived markers (TDMs) was used to design specific primer pairs. For RFLPs, sequences were downloaded from the GrainGenes database (http://wheat.pw.usda.gov/GG2/index.shtml). For TDMs, unigene sequences were downloaded from the Barley SNP Database (http://germinate.scri.ac.uk/barley_snpdb/dbStats_contig.html) (Potokina et al. 2008). For sequence-tagged site (STS) markers, the primer sequences were obtained directly from the GrainGenes database. The primer sequences of the simple sequence repeat (SSR) markers were obtained from literature (Varshney et al. 2007). Sequence for the Diversity Arrays Technology (DArT) marker ctg15632 and primers for the Cleaved Amplified Polymorphic Sequence (CAPS) marker Uni19962 have been reported elsewhere (Boyd and Horsley 2007).

#### Approach II

Conserved microsynteny between barley, rice, and *Brachypodium distachyon* was also used to generate new markers closely linked to *Rphq11* and *rphq16*. The sequences of EST-based markers mapped in the vicinity of *Rphq11* and *rphq16* were used for BLAST searches against rice and *B. distachyon* genome databases; i.e., the Rice Genome Annotation Project BLAST search (http://rice.plantbiology.msu.edu/analyses_search_blast.shtml) and the *B. distachyon* BLAST portal (http://plants.ensembl.org/Brachypodium_distachyon). Rice and *B. distachyon* gene sequences within the identified synteny blocks were in turn used for BLAST against the barley EST tentative consensus (TC) sequences from the barley TIGR Gene Indices database (http://compbio.dfci.harvard.edu/tgi/). Only barley TC sequences with a BLAST hit having an E-value ≤10^−15^ were further considered for primer design and marker development. To maximise the chance of developing markers that map in the target regions of the barley genome, only barley TC sequences having an homologous gene in the syntenic regions of both rice and *B. distachyon* were further considered for primer design and marker development.

Primers were designed using the Lasergene software (DNASTAR® 8 Inc., Madison, WI, USA). For each primer pair, a gradient PCR was performed to determine the optimal annealing temperature. Sequence-characterized amplified region (SCAR) markers were obtained by finding length polymorphism or allele-specific amplification directly after PCR on parental lines. For primers that amplified fragments of the same size in parental lines, CAPS markers were developed. The PCR products were sent for sequencing (BaseClear, Leiden, the Netherlands). SNPs were identified from the sequence obtained, using the Lasergene software. The dCAPS finder program [http://helix.wustl.edu/dcaps/dcaps.html; (Neff et al. 2002)] was then used to find discriminating restriction enzymes.

Markers developed based on TDMs, synteny, and eQTL candidate genes were named with the prefix WBE for Wageningen Barley ESTs.

## Results

### Mapping QTL for partial resistance at adult stage in S/M and OWB populations

A significant repetition × genotype effect was observed for the adult plant disease tests of both S/M and OWB mapping populations. Consequently, QTL mapping was performed for each repetition independently. In both populations and in each repetition, RLP50A showed a continuous distribution of phenotypes with transgressive segregation (Supplementary Material, Fig. S3). On Steptoe and Rec, RLP50A was always higher than on Morex and Dom, respectively, except in the first repetition of the OWB population where Rec and Dom had nearly the same RLP50A. A permutation test suggested a LOD threshold of 3 for each repetition. A QTL was declared only when its LOD profile surpassed this threshold in at least two repetitions of the same population.

Two partial resistance QTLs were mapped in S/M, on chromosomes 1H and 3H (Table 1). These two QTLs were mapped in regions where no partial resistance QTL was reported before (Supplementary Material, Fig. S4). They are designated as *Rphq22* and *Rphq23*, respectively. *Rphq22* was detected as significant (LOD >3) QTL in all three repetitions of the disease test. *Rphq23* was mapped in two repetitions of the disease test while its LOD score in the third repetition was just below the threshold. *Rphq22* explained approximately 26% of the phenotypic variation and *Rphq23* explained around 22%. For both QTLs, the resistance allele was donated by Steptoe. No QTL resistance allele was found to be contributed by Morex despite the observed transgressive segregation in the mapping population. This most probably indicates the presence of QTLs with effects too small to be detected in this experiment. The QTLs for heading date (HD) and plant height (PH) segregating in S/M were also positioned on the integrated map. *Rphq22* and *Rphq23* collocate neither with the HD nor the PH QTLs.

**Table 1:** Summary of partial resistance QTLs against barley leaf rust isolate 1.2.1 detected at adult plant stage in S/M DH mapping population

QTL	Chrom	Peak marker	cM^a^	LOD	Exp%^b^	Donor
*Rphq22*	1H	Contig8593	134.4	8.6	26.1	Steptoe
*Rphq23*	3H	Contig10370	101.9	6.1	21.7	Steptoe

The QTL features are based on the series with the highest LOD score using MAPQTL®6 (van Ooijen 2009)

^a^Peak marker position on the integrated map “Barley, Integrated, Marcel 2009”

^b^Percentage of explained phenotypic variance (MapQTL®6)

For OWB, the Pearson coefficient correlation of RLP50A between repetitions was very low (data not presented). There was no QTL identified in at least two repetitions of the disease test. In each repetition, a different unique QTL was identified, on chromosomes 2H, 5H and 7H, respectively.

In none of the three repetitions of disease tests for S/M and OWB, we detected our target QTLs *Rphq11* and *rphq16*. Therefore, it is likely that these two QTLs are effective only at seedling stage (Marcel et al. 2007b), and not at adult plant stage.

### Markers developed for *Rphq11* and *rphq16*

Twenty markers were developed that supposedly mapped on chromosome 2HL in the region of *Rphq11* flanked by the markers Bmag0125 and GBMS244 (Supplementary Material, Table S1). Among those, 16 markers mapped between the flanking markers while the other 4 markers (one CAPS and three SSR) mapped near but outside the flanked QTL interval. The 16 markers consist of two SSR, two SCAR and 12 CAPS markers. Seven of the linked markers are synteny-based markers, developed using rice and *B. distachyon* annotated genes. The rice syntenic region on chromosome 4 was identified by BLAST with Uni19962 and GBM1062 sequences. Uni19962 is homologous to Loc_Os04g47040 in rice, and GBM1062 is homologous to Loc_Os04g46820 in rice. However, there is no *B. distachyon* homologue for Uni19962 and GBM1062. Therefore the *B. distachyon* syntenic region on chromosome Bd5 was based on the rice homologue of Uni19962 and GBM1062, as well as two EST-based markers, WBE144 and WBE129, which were flanked by Uni19962 and GBM1062. WBE144 is homologous to Bradi5g17980 in *B. distachyon*, and WBE129 is homologous to Bradi5g18000 in *B. distachyon*.

For *rphq16*, 27 markers were developed that supposedly mapped on chromosome 5HL in the QTL confidence interval flanked by the markers ABG391 and GMS002 (Supplementary Material, Table S2). Among those, 18 markers mapped between the flanking markers of the QTL while the other 9 markers (1 SCAR, 5 CAPS and 3 SSR) mapped near but outside the flanked QTL interval. Three of the markers closely linked to *rphq16* are synteny-based markers. The rice syntenic region on chromosome 3 and *B. distachyon* syntenic region on chromosome Bd1 was identified by BLAST with WBE320 and GBS0408 sequences. WBE320 is homologous to Loc_Os03g63450 in rice and Bradi1g01500 in *B. distachyon*. GBS0408 is homologous to Loc_Os03g63940 in rice and Bradi1g00990 in *B. distachyon*.

### High-resolution genetic map for *Rphq11* and *rphq16*

**Fig. 1:**
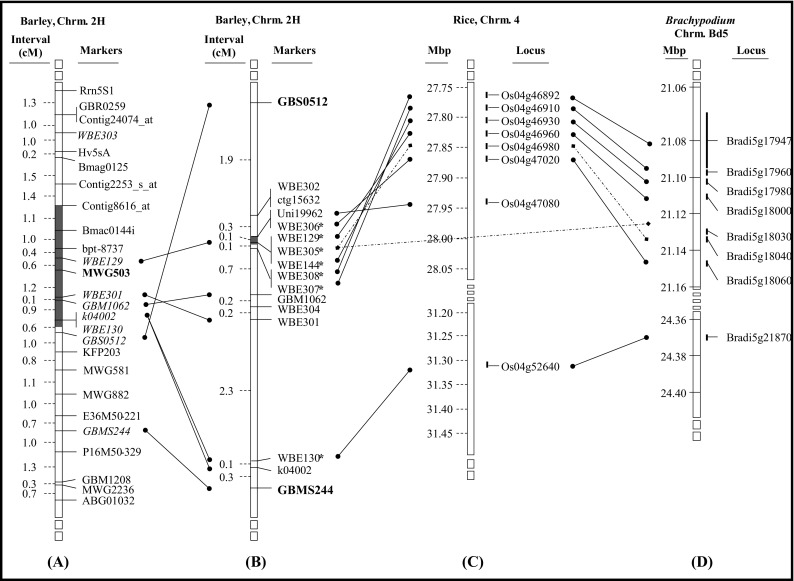
Alignment of **a** the integrated map “Marcel 2009” and **b** the high-resolution map generated in this study, at the *Rphq11* region on barley chromosome 2HL with **c** the physical map of rice chromosome 4 and **d** the physical map of *B. distachyon* chromosome Bd5. The *filled grey areas* inside chromosome* bars* indicate the position of *Rphq11*. The *bold marker* on **a** is the peak marker of *Rphq11*. The *bold markers* on **b** are the flanking markers used for recombinant screening, and the *markers with an asterisk* on **b** are synteny-based markers. The *dashed lines* show homologous sequences found between only two of the three species barley, rice and *B. distachyon*

**Fig. 2:**
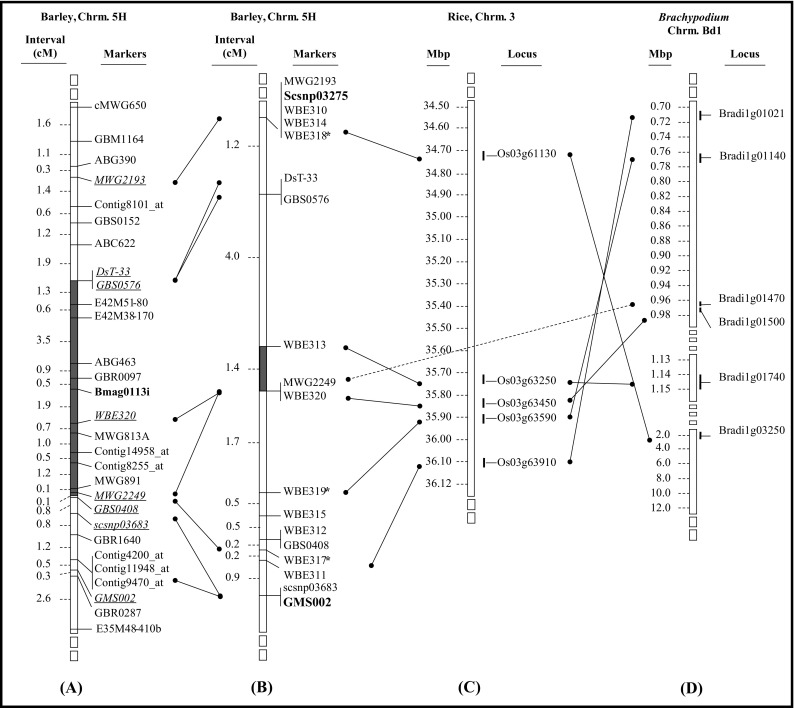
Alignment of **a** the integrated map “Marcel 2009” and **b** the high-resolution map generated in this study, at the *rphq16* region on barley chromosome 5HL with **c** the physical map of rice chromosome 3 and **d** the physical map of *B. distachyon* Bd1. The *filled grey areas* inside chromosome *bars* indicate the position of *rphq16*. The *bold marker* on **a** is the peak marker of *rphq16*. The *bold markers* on **b** are the flanking markers used for recombinant screening, and the *markers with an asterisk* on **b** are synteny-based markers. The dashed line shows a homologous sequence between barley and *B. distachyon*, which was only found in rice on another chromosome

There were 89 fixed QTL recombinants identified for *Rphq11* and 135 for *rphq16* (described in the next section). These fixed QTL recombinants were genotyped with the newly developed markers, and high-resolution genetic maps were generated (Figs. 1 and 2).

On the new high-resolution genetic map of the *Rphq11* region, the distance between markers GBS0512 and GBMS244, flanking *Rphq11*, is approximately 6 cM. Their interval on the barley integrated map [Barley, Integrated, Marcel 2009; (Aghnoum et al. 2010)] is comparable in size (5 cM). Sixteen markers were mapped in this interval, providing an average marker density of one marker per 0.4 cM. Marker positions of GBS0512, WBE301 and GBM1062 around *Rphq11* in the new-high resolution genetic map were slightly different compared to the integrated map. GBS0512 (Stein et al. 2007) and WBE301 (Potokina et al. 2008) were originally mapped in S/M, and GBM1062 in OWB (Varshney et al. 2007), which can explain the inaccuracy of their order on the integrated map. The 6-cM genetic window containing *Rphq11* is in synteny with rice chromosome 4 and *B. distachyon* chromosome Bd5. The orientation of the syntenic block delimited by Uni19962 and WBE307 in barley is inverted compared to rice and *B. distachyon*, and microsyntenic rearrangements in marker order are also observed within the block. The orientation of the syntenic block and the order of markers are perfectly conserved between rice and *B. distachyon* (Fig. 1).

On the new high-resolution genetic map of the *rphq16* region, the distance between markers scsnp03275 and GMS002, flanking *rphq16*, is approximately 11 cM. Their interval is approximately 20 cM on the barley integrated map [Barley, Integrated, Marcel 2009; (Aghnoum et al. 2010)]. The estimated 20-cM interval was based on MWG2193, WBE310 and WBE314 which shared the same position as scsnp03275 on the high-resolution genetic map because scsnp03275 was not mapped in the integrated map.

There were 18 markers mapped in this interval, providing an average marker density of one marker per 0.6 cM. Marker order at *rphq16* was in agreement with marker order on the integrated map. The 11-cM genetic window comprising *rphq16* is in synteny with rice chromosome 3 and *B. distachyon* chromosome Bd1. The orientation of this syntenic region and marker order are perfectly conserved between barley and rice but inverted in *B. distachyon* (Fig. 2).

### Fine mapping of *Rphq11* and *rphq16*

The disease test on 97 F_3_ plants segregating for *Rphq11* showed that *Rphq11* is an incompletely dominant gene, while the disease test on 52 BC_1_S_1_ plants segregating for *rphq16* shows that *rphq16* behaves predominantly as a recessive gene (Supplementary Material, Fig. S5). The RLP50S comparison between the group of plants with homozygous donor allele (AA) and the group of plants without the donor allele (BB) shows that *Rphq11* gives an approximately 21-h (11%) prolongation of latency period on the seedling leaves and *rphq16* an approximately 14-h (7%) prolongation.

Recombinant plant screening of 730 plants at F_4_ and F_5_ resulted in 89 fixed QTL recombinants at *Rphq11* representing 10 recombination intervals between all the markers mapped between GBS0512 and GBMS244. For *rphq16*, recombinant plant screening of 655 plants at BC_2_S_1_ and BC_2_S_2_ resulted in 135 fixed QTL recombinants representing 9 recombination intervals between all the markers mapped between WBE318 and scsnp03683.

**Fig. 3:**
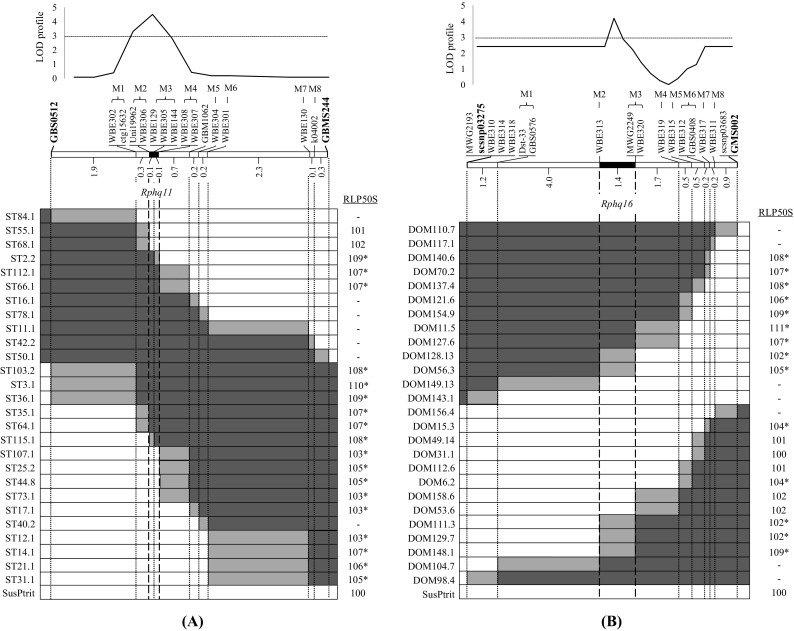
Graphical genotypes and phenotype means (RLP50S) for fixed QTL recombinants of **a** *Rphq11* and **b** *rphq16*; the phenotype means were compiled from results of the different rounds of disease test. The *white bars* represent homozygous SusPtrit. *Black bars* represent homozygous Steptoe (**a**) or Dom (**b**). *Grey bars* represent intervals where recombination took place. RLP50S values with an *asterisk* are significantly longer than the RLP50S on SusPtrit. The number between *two markers* on the chromosome *bar* indicates the recombination frequency observed. The new genetic window of *Rphq11* and *rphq16* is indicated between the *long dashed lines*

After several rounds of disease tests on a subset of the fixed QTL recombinants, *Rphq11* was fine-mapped into a genetic window of 0.2 cM flanked by markers Uni19962 and WBE306 proximally and WBE307 and WBE308 distally. Indeed, the peak of the LOD profile generated by performing QTL mapping on the fixed QTL recombinants supports this position of *Rphq11* (Fig. 3a). This is consistent with an RLP50S between 107 and 110 for fixed QTL recombinants having the *Rphq11* allele, which is always significantly higher than the RLP50S on SusPtrit. And this is consistent with an RLP50S between 101 and 107 for fixed QTL recombinants having the *rphq11* allele, which is however not always significantly shorter than those having the *Rphq11* allele and, in some cases, significantly longer than on the susceptible SusPtrit.

Similarly, *rphq16* was fine-mapped into a genetic window of 1.4 cM flanked by markers WBE313 proximally and MWG2249 and WBE320 distally. The peak of the LOD profile generated by performing QTL mapping on the fixed QTL recombinants supports this position of *rphq16* (Fig. 3b). This is consistent with RLP50S on fixed QTL recombinants having the *rphq16* allele ranging from 106 to 111, which is always significantly longer than the RLP50S on SusPtrit. And this is also consistent with RLP50S on fixed QTL recombinants having the *Rphq16* allele ranging from 100 to 105, which is not always significantly shorter than those having the *rphq16* allele and, in some cases, significantly longer than on the susceptible SusPtrit.

The refined position of *Rphq11* in a 0.2-cM interval corresponds to the syntenic region inverted between barley and rice (Fig. 1). The 0.2 cM in barley corresponds to physical distances of 161 kb with 18 annotated genes in rice and 79 kb with 9 annotated genes in *B. distachyon*. Concerning *rphq16*, its refined position of 1.4 cM in barley corresponds to physical distances of 118 kb with 20 annotated genes in rice and 188 kb with 9 annotated genes in *B. distachyon*.

## Discussion

### Plant stage-specific QTLs in S/M and OWB

The seedlings and adult plants of S/M and OWB mapping populations were challenged with *Ph*.1.2.1. None of the partial resistance QTLs that were detected at seedling stage (Marcel et al. 2007b) were also detected in any of the three repetitions of the disease test at adult plant developmental stage (this study). This indicates that *Rphq11* and *rphq16* are plant growth stage dependent and that their characterisation can only be performed at seedling stage. *Rphq22* and *Rphq23*, which were detected in this study at adult stage in S/M, are also plant growth stage dependent, since they were not detected in the earlier seedling tests. This plant stage dependence of QTLs for partial resistance against *P. hordei* was also reported for other partial resistance QTLs in this pathosystem (Qi et al. 1998; Wang et al. 2010).

Plant growth characteristics, such as heading date and plant height, may influence the resistance of plants (Klahr et al. 2007; Williams 2003). In rice, the germin-like protein 1 was demonstrated to be involved in regulating plant height and disease resistance (Banerjee and Maiti 2010). *Rphq22* and *Rphq23* did not collocate with any heading date and plant height QTLs, which suggest that the resistance conferred by these two QTLs is not a pleiotropic effect of genes affecting heading date or plant height.

### Adult plant resistance QTLs from S/M and OWB are affected by the environment

In both populations, a clear repetition × genotype effect was observed, which was especially strong in the OWB population. From the three QTLs mapped in S/M, one was identified in only one of the three repetitions. And all three QTLs mapped in OWB were identified in only one of the three repetitions. Environmental effects may be among the contributing factors for the inconsistency of QTL identification. The disease tests were carried out at different time periods of the year, and the third repetition was carried out at a different greenhouse facility.

The high morphological variation between the OWB lines may also have contributed to the inconsistency of QTLs mapped in this population. The parental lines of the OWB population have been developed by systematically crossing recessive alleles for morphological and physiological traits into one parent and dominant alleles into the other parent, maximizing the genetic, morphological and agronomic diversity segregating in the population (Costa et al. 2001). The variation in plant height may affect the uniformity of inoculum deposition. Heading date variation can also influence the result of a disease test as the sequential inoculation of different groups of lines according to their heading date may compromise uniformity and randomisation of DH lines over batches. Notably, the disease resistance of DH lines may vary based on the age of the flag leaf used. It is difficult to homogenise the disease tests for this population due to the high morphological variation between lines.

### Efficient fine mapping of *Rphq11* and *rphq16*

A QTL can be fine-mapped without the interference of other QTLs if NILs were used as starting material. The NIL development is, however, laborious and time consuming. For barley, one generation is approximately 4 months; thus, the development of a NIL with approximately 95% of its genome coming from the recurrent parent will take 2 years and 4 months to reach generation BC_4_. Another year will be necessary to obtain enough seeds of homozygous recombinant plants (i.e. sub-NILs) to allow fine mapping. In total, about 3 years and 4 months are needed to fine-map a QTL to a certain genetic window. One way to shorten this procedure is by exploiting individual lines from recombinant inbred lines (RILs), DH lines, chromosome segment substitution lines (CSSLs) and backcross inbred lines (BILs) (Gao et al. 2004; Liu and Bai 2010; Zhang et al. 2011). Effectively, individual line(s) which contain only the resistance allele of the targeted QTL can be crossed with a susceptible parent in order to generate recombinant plants that will be used for fine mapping. However, the construction of RILs, CSSL and BILs is as laborious and time consuming as NIL and sub-NIL development.

Tuinstra et al. (1997) proposed a method to identify QTL-NILs from populations of advanced generation inbreds, usually F_5_ or F_6_ generation inbred lines. Individuals from one inbred family segregating for a selected marker linked to the QTL of interest will be nearly isogenic allowing to confirm the phenotypic effect of the QTL and to screen for recombinants at this QTL. The fine mapping strategy followed in our study aimed at identifying recombinants in early plant material developed to produce QTL-NILs. The material was first selected to carry the targeted QTL in heterozygous condition and susceptibility alleles at other resistance QTLs in homozygous condition. This strategy took four to five generations to obtain fixed QTL recombinants. This way, it became possible to fine-map a QTL in less than 2 years. In parallel, the NILs of the targeted QTLs were developed, which allowed the confirmation of the effect of *Rphq11* and *rphq16* in an isogenic background (data not shown).

The position of *Rphq11* has been narrowed down to a genetic window of 0.2 cM (1460 gametes scored) and *rphq16* to a genetic window of 1.4 cM (1310 gametes scored). The fixed QTL recombinants were not monitored for the presence of donor genome outside the regions of the targeted QTLs. Theoretically, the plant materials (i.e. F_3_) used to fine-map *Rphq11* should have approximately 50% of donor genome. For *rphq16*, the plant materials (i.e. BC_2_S_1_) may still have approximately 13% of donor genome. Consequently, previously undetected minor effect QTLs for resistance may still be present and even segregate in the material used to fine-map *Rphq11* and *rphq16*. As a result of this heterogeneous genetic background, we may expect genetic and hence phenotypic noise in determining the QTL position and assessment of its effect. This may explain that several of the fixed QTL recombinants carrying the susceptibility allele at the target QTL give a significantly longer latency period than the susceptible line SusPtrit used for crossing.

Nevertheless, it was still possible to dissect the position of *Rphq11* and *rphq16* as the RLP50 range between fixed QTL recombinants carrying the susceptibility allele and fixed QTL recombinants carrying the resistance allele at the corresponding QTL was nearly distinct (i.e. RLP50S was 101–107 versus 107–110 for *Rphq11*, and 100–105 versus 106–111 for *rphq16*). Moreover, the positions of *Rphq11* and *rphq16* were supported by mapping QTLs on the high-resolution map. The peak marker of *Rphq11* became WBE129. The peak marker for *rphq16* became MWG2249/WBE320, which is one of the markers flanking the window. Note that MWG2249/WBE320 has the second highest LOD score. The peak LOD score for *rphq16* was in a marker interval. The new small genetic window of *Rphq11* and *rphq16* remains consistent with the position of their peak marker previously identified by Marcel et al. (2007b) (Figs. 1 and 2). The position of *Rphq11* is in agreement with the eQTL mapping performed in the S/M population by Chen et al. (2010). The authors mapped eQTLs with measures of transcript abundance obtained in the S/M population 18 h after inoculation with *P. hordei* isolate 1.2.1. They analysed the correlation between the identified eQTL and the pQTL including *Rphq11*. They identified 54 eQTLs located in the confidence interval of *Rphq11*, and 6 genes were proposed as candidate genes for *Rphq11*. Of these six, ‘Unigene2453’ encoding a phospholipid hydroperoxide glutathione peroxidase (PHGPx) was considered the strongest candidate for *Rphq11* (Chen et al. 2010). Interestingly, the marker developed on Unigene2453, WBE129, indeed was the peak marker of *Rphq11* located within the refined 0.2-cM position of this pQTL. The six most strong candidates listed by Chen et al. (2010) all have homologues in rice, but out of those six, only the gene corresponding to PHGPx (Os04g46960) is located in the syntenic region corresponding with the barley WBE306 to WBE307 interval.

*Rphq11* and *rphq16* are the second and third fine-mapped QTLs for barley partial resistance to leaf rust. The fine mapping strategy followed in this study has proven efficient to fine-map these two QTLs with a slightly smaller effect on the resistance level than *Rphq2*. The latter gene explained 50% of the phenotypic variance and was fine-mapped in another study (Marcel et al. 2007a). However, in order to fine-map smaller-effect QTLs, it would probably be necessary to reduce the noise caused by genetic background by starting the fine mapping process at BC_3_ or at even later backcross generations as suggested by Yang et al. (2012).

### Identification of candidate genes for *Rphq11* and *rphq16* based on synteny between barley, rice and *B. distachyon*

The genetic window of *Rphq11* is syntenic with rice chromosome 4, in agreement with Pourkheirandish et al. (2007), and with *B. distachyon* chromosome Bd5 (Mayer et al. 2011). The orientation of the syntenic region corresponding to *Rphq11* is conserved between rice and *B. distachyon* but inverted in barley. The size of the *Rphq11* syntenic region in rice is approximately 161 kb, with 18 annotated genes. In *B. distachyon*, the size is approximately 79 kb with nine annotated genes. None of the genes found in this region possess a nucleotide-binding site (NBS) or a leucine-rich repeat (LRR) domain commonly found in major resistance genes (DeYoung and Innes 2006). However, several genes belong to gene families previously shown to be involved in resistance in other plant pathogen systems. These genes include an actin-depolymerizing factor (Wang et al. 2013), a glutathione peroxidase (Lamb and Dixon 1997) and glycosyltransferases (Langlois-Meurinne et al. 2005; von Saint et al. 2011). Homologues of all those genes are found in the syntenic region of both rice and *B. distachyon*.

Glutathione peroxidase is the most promising candidate gene for *Rphq11*. The peak marker for *Rphq11* on the high-resolution genetic map is WBE129, which has been developed on the candidate gene Unigene2453 encoding for the phospholipid hydroperoxide glutathione peroxidase (PHGPx). This PHGPx gene has also been identified as the strongest candidate to explain *Rphq11* by Chen et al. (2010), because it was detected as a high-LOD *cis*-regulated expressed QTL with significantly different transcript abundances between Steptoe and Morex. Out of the six strongest candidate genes emerging from the work of Chen et al. (2010), it is the only gene located in the syntenic rice interval (see above). As this PHGPx gene is conserved across rice, *B. distachyon* and barley, it may also have a conserved function in defence against pathogens across plant species. In rice, the expression of rice PHGPx homologue—*Os*PHGPx—is induced by infection with *Magnaporthe grisea* (Agrawal et al. 2002). The tomato PHGPx homologue—LePHGPx—also confers resistance, against *Botrytis cinerea*, when stably expressed in tobacco (Chen et al. 2004).

The genetic window of *rphq16* is syntenic with rice chromosome 3, in agreement with Close et al. (2009), and *B. distachyon* chromosome Bd1 (Mayer et al. 2011). The orientation of the syntenic region corresponding to *rphq16* is conserved between barley and rice, but it is inverted in *B. distachyon*. The size of the syntenic region is approximately 118 kb with 20 annotated genes in rice and 188 kb with 9 annotated genes in *B. distachyon*. Several of these genes belong to gene families involved in resistance in other plant pathogen systems, including an oxidoreductase (Montesano et al. 2003), an aspartokinase (Stuttmann et al. 2011) and a proteasome subunit (Yao et al. 2012), which are conserved between rice and *B. distachyon*. There is also a glutathione S-transferase (Dean et al. 2005) and a transcription factor BTF3 (Huh et al. 2012) found only in the rice syntenic region, as well as a protein kinase C (Subramaniam et al. 1997), protein tyrosine phosphatases (He et al. 2012), glycosyltransferases (Langlois-Meurinne et al. 2005) and a NBS-LRR gene (Loutre et al. 2009) found only in the *B. distachyon* syntenic region. There is no favourite candidate gene in the interval for the moment.

### Feasibility of map-based cloning *Rphq11* and *rphq16*

*Rphq11* or *rphq16* prolongs the latency period of *P. hordei* isolate 1.2.1. by 12 h in comparison to SusPtrit, which proved to be sufficient to differentiate plants with the resistance allele from those with the susceptibility allele at each QTL. In agreement with this observation, the effect of *Rphq11* and of *rphq16* was confirmed in isogenic lines (data not shown). It allowed fine mapping *Rphq11* and *rphq16* to barley regions of 0.2 and 1.4 cM, respectively, following a time-efficient strategy. Even though there is phenotypic noise, it was still possible to dissect the position of *Rphq11* and *rphq16*. *Rphq11* was mapped in a high recombination rate region (1.1 Mb/cM) of approximately 220 kb from barley chromosome 2H and *rphq16* in a very high recombination rate region (0.2–0.9 Mb/cM) of approximately 200–900 kb from barley chromosome 5H (Künzel et al. 2000). This offers good prospects for the map-based cloning of the gene(s) underlying these two QTLs.

Many QTLs for partial resistance were mapped in barley against barley leaf rust, but the underlying genes have not been identified so far. *Rphq2* has previously been fine-mapped to a genetic interval of 0.11 cM (Marcel et al. 2007a), encompassing a barley region of approximately 190 kb. This area comprises about 12 genes in partially resistant Vada, of which 7 do not correspond to any gene in the susceptible parent SusPtrit (Yeo et al. 2015). The precise mapping of *Rphq11* and *rphq16* is a new step towards the understanding of the genetic basis of partial resistance to barley leaf rust. The sequenced Morex genome (The International Barley Genome Sequencing Consortium 2012) can be the reference for constructing the physical maps of Steptoe and Dom and for identifying candidate genes for *Rphq11* and *rphq16.* If the genes for *Rphq11* and *rphq16* are not present in Morex, bacterial artificial chromosomes (BACs) for Steptoe and Dom should be constructed in order to build physical maps and identify candidate genes. The identification of the gene(s) for *Rphq2*, *Rphq11* and *rphq16* will reveal if the genes for barley partial resistance belong to the same or different gene families.

## Electronic supplementary material

ESM 1 (DOCX 144 kb).

ESM 2 (DOCX 144 kb).

ESM 3 (DOCX 134 kb).

ESM 4 (DOCX 134 kb).

ESM 5 (DOCX 27 kb).

ESM 6 (DOCX 20 kb).

ESM 7 (DOCX 22 kb).

ESM 8 (DOCX 418 kb.)
