# Supplementary material for: High-resolution mapping of genes involved in plant stage-specific partial resistance of barley to leaf rust
Source: Mol Breed. 2017 Mar 16;37(4):45. doi: 10.1007/s11032-017-0624-x (PMC5352788; doi:10.1007/s11032-017-0624-x)
Supplement: Supplementary file 1 — (DOCX 144 kb). [file 11032_2017_624_MOESM1_ESM.docx]

*Figure S2*. Rounds of disease test with fixed QTL-recombinants for (A) *Rphq11* and (B) *rphq16*. The graphical genotypes represent the fixed QTL-recombinants tested in each round. The white bars represent homozygous SusPtrit; the black bars represent homozygous Steptoe (A) or Dom (B); and the grey bars represent intervals where recombination took place. Molecular markers in bold are the flanking markers used after preliminary fine-mapping. M – New markers obtained (Figure 3).
