# Supplementary material for: High-resolution mapping of genes involved in plant stage-specific partial resistance of barley to leaf rust
Source: Mol Breed. 2017 Mar 16;37(4):45. doi: 10.1007/s11032-017-0624-x (PMC5352788; doi:10.1007/s11032-017-0624-x)
Supplement: Supplementary file 3 — (DOCX 134 kb). [file 11032_2017_624_MOESM3_ESM.docx]

*Figure S4.* The map position of QTLs for partial resistance to *P. hordei* mapped in five barley mapping populations (including the QTLs detected in this study) on the integrated map, Marcel 2009. The length of the QTL bars corresponds approximately to the rMQM one LOD confidence interval and the extended lines from the QTL bars corresponds approximately to the rMQM two LOD confidence interval. QTLs with an asterisk are QTLs detected in this study. The black bars within chromosome bars correspond to plant height QTLs and grey bars to heading date QTLs. Numbers on the left side of chromosome bars show the distance in cM according to Kosambi.
