# Supplementary material for: High-resolution mapping of genes involved in plant stage-specific partial resistance of barley to leaf rust
Source: Mol Breed. 2017 Mar 16;37(4):45. doi: 10.1007/s11032-017-0624-x (PMC5352788; doi:10.1007/s11032-017-0624-x)
Supplement: Supplementary file 5 — (DOCX 27 kb). [file 11032_2017_624_MOESM5_ESM.docx]

*Figure S5*. Histograms of the averaged RLP50S of (A) F_3_ seedlings segregrating for *Rphq11* and (B) BC_1_S_1_ seedlings segregating for *rphq16*. ‘A’ is the allele from Steptoe/ Dom and ‘B’ is the allele from SusPtrit. Same letters above the bars indicate that the variance do not differ significantly according to the unbalanced one-way ANOVA analysis.
