# Supplementary material for: High-resolution mapping of genes involved in plant stage-specific partial resistance of barley to leaf rust
Source: Mol Breed. 2017 Mar 16;37(4):45. doi: 10.1007/s11032-017-0624-x (PMC5352788; doi:10.1007/s11032-017-0624-x)
Supplement: Supplementary file 6 — (DOCX 20 kb). [file 11032_2017_624_MOESM6_ESM.docx]

| **Table S1** List of molecular markers mapped at *Rphq11*. | | | | | | |
| --- | --- | --- | --- | --- | --- | --- |
| Name | Type | Chrom. | Restriction enzyme | Tm  (^o^C) | Primer sequences (5’-3’) | Source^b^ |
| Ctg15632 | CAPS | 2H | MboII | 58 | *F:* TGGCAAATGACGGGGCACTAAAAC | Boyd et al. (2007) |
|  |  |  |  |  | *R:* AACCGGCCTACAGATCGCAACCTT |  |
| GBM1062 | SSR | 2H |  |  | Confidential | Li et al. (2003) |
|  |  |  |  |  |  |  |
| GBMS244 | SSR | 2H |  |  | Confidential | Thiel et al. (2003) |
|  |  |  |  |  |  |  |
| GBS0512 | CAPS | 2H | AciI | 58 | *F:* CCACATGCTGCGGAGGT | Stein et al. (2007) |
|  |  |  |  |  | *R:* CGTTGAGGATGATGCTGAGG |  |
| k04002 | SCAR | 2H |  | 60 | *F:* GACACAGGACCTGAAGCACA | Hori et al. (2005) |
|  |  |  |  |  | *R:* CGGCAGGCTCTACTATGAGG |  |
| Uni19962 | CAPS | 2H | MseI | 58 | *F:* GTCCCACATCACTGCACATC | Boyd et al. (2007 ) |
|  |  |  |  |  | *R:* CAGTCGCAGAAGTTACTGAAG |  |
| WBE129 | CAPS | 2H | HpyCHY4IV | 58 | *F:* CCCCCAAACTCCCAACT | Rice synteny |
|  |  |  |  |  | *R:* CTCCAGCCAGCAGGTCTAA |  |
| WBE130 | dCAPS | 2H | XapI | 58 | *F:* CTCGTATGTTGTGTGGAATTGTGAGC-CCAATCTTAATCCTAAGATCTCGAA | Rice synteny |
|  |  |  |  |  | *R:* GGTCTCCCAGCTAAAGTCTCC |  |
| WBE144 | CAPS | 2H | BsrI | 58 | *F:* GAGGCCCTTATCATTCTGTTGTCC | Rice synteny |
|  |  |  |  |  | *R:* ATGCTGGCGCGTTTTTGGGTATG |  |
| WBE301 | SCAR | 2H |  | 65 | *F:* TCGATGAGCGGATGGGTAAGGTAT | Potokina et al. (2008) |
|  |  |  |  |  | *R:* ATTCCCAGCTGCCCAGTGTTTCT |  |
| WBE302 | CAPS | 2H | Tsp4CI | 65 | *F:* ATGATCTTCGCCCTCGTCTACTGC | Potokina et al. (2008) |
|  |  |  |  |  | *R:* TGGTCTTGAATGGGATCGCTCTGA |  |
| WBE304 | CAPS | 2H | SacII | 65 | *F:* AGCTAGCTGTTGGGCGTGAAAATC | Potokina et al. (2008) |
|  |  |  |  |  | *R:* CAAGGGGGTGGAGGAGGAAGAAGT |  |
| WBE305 | CAPS | 2H | MwoI | 65 | *F:* CCGTCCCGTCACCCGAGTCC | Rice synteny |
|  |  |  |  |  | *R:* TCAGGCCTTCCAGTAGCGAGTTCC |  |
| WBE306 | CAPS | 2H | NdeI | 65 | *F:* CGGGGGCGCCTCCTCTACTC | Rice synteny |
|  |  |  |  |  | *R:* GTCCGGGTCATCATCTTCCACAAC |  |
| WBE307 | CAPS | 2H | SduI | 65 | *F:* GGCGCTCCGTGCAAAGAAGA | Rice synteny |
|  |  |  |  |  | *R:* GGAGACGAGGAGCAAAAGACACAA |  |
| WBE308 | CAPS | 2H | ClaI | 65 | *F:* CTGAGCCTGGGAAACAAAGTCG | Rice synteny |
|  |  |  |  |  | *R:* CAGCGCTGATGCAACAATAGGAT |  |
| Bmac0216a | SSR | 2H |  |  | *F:* GTACTATTCTTTGCTTGGGC | Ramsay et al. (2000) |
|  |  |  |  |  | *R:* ATACACATGTGCAAAACCATA |  |
| Bmag0125a | SSR | 2H |  |  | *F:* AATTAGCGAGAACAAAATCAC | Ramsay et al. (2000) |
|  |  |  |  |  | *R:* AGATAACGATGCACCACC |  |
| GBM1440a | SSR | 2H |  |  | *F:* CTACCGAGCTCCTCCTCCTC | Marcel et al. (2007) |
|  |  |  |  |  | *R:* GGCCTCCTTCTTGTCGTAGA |  |
| scsnp06130a | CAPS | 2H | HinfI | 56 | *F:* GACGTCCCTCGCGTAAATGG | Rostok et al. (2005) |
|  |  |  |  |  | *R:* TTGGCCGGGAACTTATGGTG |  |
| ^a^ The markers which were mapped near but outside the flanked QTL interval  ^b^ The references for CAPS and SCAR markers give the origin of the sequences obtained for marker development. | | | | | | |
