# Supplementary material for: High-resolution mapping of genes involved in plant stage-specific partial resistance of barley to leaf rust
Source: Mol Breed. 2017 Mar 16;37(4):45. doi: 10.1007/s11032-017-0624-x (PMC5352788; doi:10.1007/s11032-017-0624-x)
Supplement: Supplementary file 7 — (DOCX 22 kb). [file 11032_2017_624_MOESM7_ESM.docx]

| **Table S2** List of molecular markers mapped at *rphq16*. | | | | | | |
| --- | --- | --- | --- | --- | --- | --- |
| Name | Type | Chrom. | Restriction enzyme | Tm  (^o^C) | Primer sequences (5’-3’) | Source^b^ |
| DsT-33 | SCAR | 5H |  | 45 | *F:* GCACACATATTATCATGAAAAAGAGC | BarleyWorld.org |
|  |  |  |  |  | *R:* ACCCCAAATGAGTTTCGATG |  |
| GBS0408 | CAPS | 5H | MseI | 56 | *F:* ATGCCACCCCTTATGAATCCT | Stein et al. 2007 |
|  |  |  |  |  | *R:* TTGCCCGTTGAAAAGTCCA |  |
| GBS0576 | CAPS | 5H | BspLI | 56 | *F:* GTCCGGGCACAAGAACCTC | Stein et al. 2007 |
|  |  |  |  |  | *R:* GGCTGGGCATCATCCTCAA |  |
| GMS002 | SSR | 5H |  |  | *F:* CCGACAACATGCTATGAAGC | Struss and Plieske (1998) |
|  |  |  |  |  | *R:* CTGCAGCAAATACCCATGTG |  |
| MWG2193 | CAPS | 5H | AluI | 56 | *F:* CAAACCCTTGAGGTCAGTTGC | Graner et al. (1991) |
|  |  |  |  |  | *R:* TCAGCTCTAAGATGCAGCACG |  |
| MWG2249 | CAPS | 5H | DdeI | 56 | *F:* GGCATGTGAGGGAAGCAATGG | Graner et al. (1991) |
|  |  |  |  |  | *R:* TGGAGAAGAACGTGTGGGTCG |  |
| scsnp03275 | CAPS | 5H | BglII | 65 | *F:* AACGGCCAGGCTATAACCATCACA | Rostoks et al. (2005) |
|  |  |  |  |  | *R:* CGGCGGCTTCATCAATTTCACTAA |  |
| scsnp03683 | CAPS | 5H | HpyCHY4IV | 56 | *F:* CAACGGCGCCACCTTCTACT | Rostoks et al. (2005) |
|  |  |  |  |  | *R:* CACATACCCCACTGCCATGC |  |
| WBE310 | SCAR | 5H |  | 65 | *F:* GGCGCTTTTGGTTTTCCTGA | Potokina et al. (2008) |
|  |  |  |  |  | *R:* CGGCCTGGTATAATTAAGAGTGTG |  |
| WBE311 | SCAR | 5H |  | 65 | *F:* CCAGAAAGGCGAGGAAGG | Potokina et al. (2008) |
|  |  |  |  |  | *R:* TCGGATTATTGCACACCAGAAAAC |  |
| WBE312 | CAPS | 5H | MseI | 58 | *F:* TGTGCCGTGTTATAATGGGGAATG | Potokina et al. (2008) |
|  |  |  |  |  | *R:* CACAAAATCGGGCCTGCTTATCTT |  |
| WBE313 | CAPS | 5H | MwoI | 58 | *F:* TGCCGAGTCGCCTAACCATA | Potokina et al. (2008) |
|  |  |  |  |  | *R:* TCAACAACTACCTGCCAAATACCA |  |
| WBE314 | CAPS | 5H | SphI | 65 | *F:* CCAGGGAATTACCAGGGAGACA | Potokina et al. (2008) |
|  |  |  |  |  | *R:* TGAAGCCGACAACAAAAACAGG |  |
| WBE315 | CAPS | 5H | HinfI | 65 | *F:* CCCCCTTCGCCGGCTTCTCAACC | Potokina et al. (2008) |
|  |  |  |  |  | *R:* ATTCACAAAGCGCCGGCACACCAG |  |
| WBE317 | CAPS | 5H | AcyI | 65 | *F:* ATCCCAGCCGACAGCATCC | Rice synteny |
|  |  |  |  |  | *R:* GAGAGCAGGCACCCGCATAG |  |
| WBE318 | CAPS | 5H | Hin1II | 65 | *F:* ACGGTGGTGGTGGTGGTCA | Rice synteny |
|  |  |  |  |  | *R:* GCCCGCAGCGTCTCGTAG |  |
| WBE319 | CAPS | 5H | HhaI | 65 | *F:* GATGGGTAGGCTTAAGCAGAAACT | Rice synteny |
|  |  |  |  |  | *R:* AACGCGCCTAACACAAACTCCTAC |  |
| WBE320 | CAPS | 5H | MseI | 58 | *F:* CCCCCGGCTGGTGTGGA | Potokina et al. (2008) |
|  |  |  |  |  | *R:* CAGCTGTGGCGTGATGTATTTGTA |  |
| ABC622^a^ | CAPS | 5H | AluI | 65 | *F:* AGGGAAGGGCTGCAAACTGTA | Rostoks et al. (2005) |
|  |  |  |  |  | *R:* ACCAACTGATCGCTGCCTGTGTAT |  |
| ABG390 ^a^ | CAPS | 5H | AluI | 56 | *F:* TGTTCCCAGCATTTGAACAG | Rostoks et al. (2005) |
|  |  |  |  |  | *R:* CGGCAATCCTAATTTTTGGA |  |
| ABG391 ^a^ | CAPS | 5H | AluI | 56 | *F:* GCAAGTGCACTGCTGTACAA | Rostoks et al. (2005) |
|  |  |  |  |  | *R:* TGTTCTCGTACCATGACTTC |  |
| CMWG650 ^a^ | CAPS | 5H | Hin1II | 56 | *F:* ATGCCTGGGTACAAAAATCAAATG | Stein et al. 2007 |
|  |  |  |  |  | *R:* TCACCCAGCCTACCAAAATAACAG |  |
| GMS001 ^a^ | SSR | 5H |  |  | *F:* CTGACCCTTTGCTTAACATGC | Struss and Plieske (1998) |
|  |  |  |  |  | *R:* TCAGCGTGACAAACAATAAAGG |  |
| scsnp00635 ^a^ | CAPS | 5H | HinfI | 65 | *F:* TGAGCAGCCGTGTCAGCTTC | Rostoks et al. (2005) |
|  |  |  |  |  | *R:* AAACATTGGATTGGGCACGC |  |
| scsnp07825 ^a^ | SCAR | 5H |  | 65 | *F:* GGCGCGGCGGACTGACAAG | Rostoks et al. (2005) |
|  |  |  |  |  | *R:* GTGGTGCTGCGACGAGGAGACG |  |
| scssr03907 ^a^ | SSR | 5H |  |  | *F:* CTCCCATCACACCATCTGTC | Ramsay et al. (2004) |
|  |  |  |  |  | *R:* GACATGGTTCCCTTCTTCTTC |  |
| scssr09041 ^a^ | SSR | 5H |  |  | *F:* CATGTCAGTGGGGTTCTAGC | Ramsay et al. (2004) |
|  |  |  |  |  | *R:* TCTACTTGGACCTGCTGACC |  |
| ^a^ The markers which were mapped near but outside the flanked QTL interval  ^b^ The references for CAPS and SCAR markers give the origin of the sequences obtained for marker development. | | | | | | |
