# Supplementary material for: High-resolution mapping of genes involved in plant stage-specific partial resistance of barley to leaf rust
Source: Mol Breed. 2017 Mar 16;37(4):45. doi: 10.1007/s11032-017-0624-x (PMC5352788; doi:10.1007/s11032-017-0624-x)
Supplement: Supplementary file 8 — (DOCX 418 kb.) [file 11032_2017_624_MOESM8_ESM.docx]

**List of supporting information, Figures**

*Figure S1.* Procedure for fine-mapping (A) *Rphq11* and (B) *rphq16* in parallel with their NIL development programs.

*Figure S2.* Rounds of disease test with fixed QTL-recombinants for (A) *Rphq11* and (B) *rphq16*. The graphical genotypes represent the fixed QTL-recombinants tested in each round. The white bars represent homozygous SusPtrit; the black bars represent homozygous Steptoe (A) or Dom (B); and the grey bars represent intervals where recombination took place. Molecular markers in bold are the flanking markers used after preliminary fine-mapping. M – New markers obtained (Figure 3).

*Figure S3.* Frequency distribution of the relative latency period at the adult plant stage (RLP50A) within S/M and OWB populations. Values of the parental lines are indicated by an arrow. (A) S/M first repetition (B) S/M second repetition (C) S/M third repetition (D) OWB first repetition (E) OWB second repetition (F) OWB third repetition. Values within brackets indicate the range of RLP50A for each parental line.

*Figure S4.* The map position of QTLs for partial resistance to *P. hordei* mapped in five barley mapping populations (including the QTLs detected in this study) on the integrated map, Marcel 2009. The length of the QTL bars corresponds approximately to the rMQM one LOD confidence interval and the extended lines from the QTL bars corresponds approximately to the rMQM two LOD confidence interval. QTLs with an asterisk are QTLs detected in this study. The black bars within chromosome bars correspond to plant height QTLs and grey bars to heading date QTLs. Numbers on the left side of chromosome bars show the distance in cM (based on Kosambi).

*Figure S5.* Histograms of the averaged RLP50S of (A) F_3_ seedlings segregrating for *Rphq11* and (B) BC_1_S_1_ seedlings segregating for *rphq16*. ‘A’ is the allele from Steptoe/ Dom and ‘B’ is the allele from SusPtrit. Same letters above the bars indicate that the variance do not differ significantly according to the unbalanced one-way ANOVA analysis.

**(Additional recombinant screen)**

**F**

**4**

**F**

**5**

**X**

**X**

**F**

**3**

**(Selected heterozygous plants)**

**(Fixed QTL**

**-**

**recombinant screen,**

**a**

**nd fine**

**-**

**mapping)**

(

**A**

)

**(Selected F**

**2**

**plants)**

***Rphq11***

**NIL**

**Steptoe x**

**SusPtrit**

**F**

**1**

**F**

**2**

**x**

**SusPtrit**

**F**

**2**

**BC**

**1**

**x**

**SusPtrit**

**... to**

**F**

**2**

**BC**

**5**

**SusPtrit**

**-**

**NIL**

***Rphq11***

**fine**

**-**

**mapping**

**F**

**2**

**F**

**4**

**F**

**3**

**(Recombinant screen)**

**(Fixed**

**QTL**

**-**

**recombinant**

**screen, preliminary fine**

**-**

**mapping)**

**X**

**F**

**2**

**BC**

**2**

**x**

**SusPtrit**

**X**

**X**

**X**

**(Selected BC**

**1**

**plants)**

**(Selected BC**

**2**

**plants)**

**BC**

**2**

**BC**

**2**

**S**

**1**

**BC**

**2**

**S**

**2**

**X**

**X**

**BC**

**2**

**x**

**SusPtrit**

***rphq16***

**NIL**

**Dom x**

**SusPtrit**

**F**

**1**

**x**

**SusPtrit**

**BC**

**1**

**x**

**SusPtrit**

**BC**

**3**

**x**

**SusPtrit**

**SusPtrit**

**-**

**NIL**

**X**

**BC**

**1**

**BC**

**1**

**S**

**1**

**BC**

**1**

**S**

**2**

**X**

**X**

**(Recombinant screen)**

**(Additional recombinant screen)**

**(Fixed QTL**

**-**

**recombinant screen,**

**and preliminary fine**

**-**

**mapping)**

***rphq16***

**fine**

**-**

**mapping**

**(Fixed QTL**

**-**

**recombinant screen**

**and fine**

**-**

**mapping)**

**... to BC**

**6**

(

**B**

)

*Figure S1.* Procedure for fine-mapping (A) *Rphq11* and (B) *rphq16* in parallel with their NIL development programs

*Figure S2*. Rounds of disease test with fixed QTL-recombinants for (A) *Rphq11* and (B) *rphq16*. The graphical genotypes represent the fixed QTL-recombinants tested in each round. The white bars represent homozygous SusPtrit; the black bars represent homozygous Steptoe (A) or Dom (B); and the grey bars represent intervals where recombination took place. Molecular markers in bold are the flanking markers used after preliminary fine-mapping. M – New markers obtained (Figure 3).

*Figure S3.* Frequency distribution of the relative latency period at the adult plant stage (RLP50A) within S/M and OWB populations. Values of the parental lines are indicated by an arrow. (A) S/M first repetition (B) S/M second repetition (C) S/M third repetition (D) OWB first repetition (E) OWB second repetition (F) OWB third repetition. Values within brackets indicate the range of RLP50A for each parental line.

*Figure S4.* The map position of QTLs for partial resistance to *P. hordei* mapped in five barley mapping populations (including the QTLs detected in this study) on the integrated map, Marcel 2009. The length of the QTL bars corresponds approximately to the rMQM one LOD confidence interval and the extended lines from the QTL bars corresponds approximately to the rMQM two LOD confidence interval. QTLs with an asterisk are QTLs detected in this study. The black bars within chromosome bars correspond to plant height QTLs and grey bars to heading date QTLs. Numbers on the left side of chromosome bars show the distance in cM according to Kosambi.

*Figure S5*. Histograms of the averaged RLP50S of (A) F_3_ seedlings segregrating for *Rphq11* and (B) BC_1_S_1_ seedlings segregating for *rphq16*. ‘A’ is the allele from Steptoe/ Dom and ‘B’ is the allele from SusPtrit. Same letters above the bars indicate that the variance do not differ significantly according to the unbalanced one-way ANOVA analysis.

**List of supporting information, Tables**

**Table S1** List of molecular markers mapped at *Rphq11*.

**Table S2** List of molecular markers mapped at *rphq16*.

**Table S3** List of molecular markers mapped at *Rphq15* and *Rphq17*.

| **Table S1** List of molecular markers mapped at *Rphq11*. | | | | | | |
| --- | --- | --- | --- | --- | --- | --- |
| Name | Type | Chrom. | Restriction enzyme | Tm  (^o^C) | Primer sequences (5’-3’) | Source^b^ |
| Ctg15632 | CAPS | 2H | MboII | 58 | *F:* TGGCAAATGACGGGGCACTAAAAC | Boyd et al. (2007) |
|  |  |  |  |  | *R:* AACCGGCCTACAGATCGCAACCTT |  |
| GBM1062 | SSR | 2H |  |  | Confidential | Li et al. (2003) |
|  |  |  |  |  |  |  |
| GBMS244 | SSR | 2H |  |  | Confidential | Thiel et al. (2003) |
|  |  |  |  |  |  |  |
| GBS0512 | CAPS | 2H | AciI | 58 | *F:* CCACATGCTGCGGAGGT | Stein et al. (2007) |
|  |  |  |  |  | *R:* CGTTGAGGATGATGCTGAGG |  |
| k04002 | SCAR | 2H |  | 60 | *F:* GACACAGGACCTGAAGCACA | Hori et al. (2005) |
|  |  |  |  |  | *R:* CGGCAGGCTCTACTATGAGG |  |
| Uni19962 | CAPS | 2H | MseI | 58 | *F:* GTCCCACATCACTGCACATC | Boyd et al. (2007 ) |
|  |  |  |  |  | *R:* CAGTCGCAGAAGTTACTGAAG |  |
| WBE129 | CAPS | 2H | HpyCHY4IV | 58 | *F:* CCCCCAAACTCCCAACT | Rice synteny |
|  |  |  |  |  | *R:* CTCCAGCCAGCAGGTCTAA |  |
| WBE130 | dCAPS | 2H | XapI | 58 | *F:* CTCGTATGTTGTGTGGAATTGTGAGC-CCAATCTTAATCCTAAGATCTCGAA | Rice synteny |
|  |  |  |  |  | *R:* GGTCTCCCAGCTAAAGTCTCC |  |
| WBE144 | CAPS | 2H | BsrI | 58 | *F:* GAGGCCCTTATCATTCTGTTGTCC | Rice synteny |
|  |  |  |  |  | *R:* ATGCTGGCGCGTTTTTGGGTATG |  |
| WBE301 | SCAR | 2H |  | 65 | *F:* TCGATGAGCGGATGGGTAAGGTAT | Potokina et al. (2008) |
|  |  |  |  |  | *R:* ATTCCCAGCTGCCCAGTGTTTCT |  |
| WBE302 | CAPS | 2H | Tsp4CI | 65 | *F:* ATGATCTTCGCCCTCGTCTACTGC | Potokina et al. (2008) |
|  |  |  |  |  | *R:* TGGTCTTGAATGGGATCGCTCTGA |  |
| WBE304 | CAPS | 2H | SacII | 65 | *F:* AGCTAGCTGTTGGGCGTGAAAATC | Potokina et al. (2008) |
|  |  |  |  |  | *R:* CAAGGGGGTGGAGGAGGAAGAAGT |  |
| WBE305 | CAPS | 2H | MwoI | 65 | *F:* CCGTCCCGTCACCCGAGTCC | Rice synteny |
|  |  |  |  |  | *R:* TCAGGCCTTCCAGTAGCGAGTTCC |  |
| WBE306 | CAPS | 2H | NdeI | 65 | *F:* CGGGGGCGCCTCCTCTACTC | Rice synteny |
|  |  |  |  |  | *R:* GTCCGGGTCATCATCTTCCACAAC |  |
| WBE307 | CAPS | 2H | SduI | 65 | *F:* GGCGCTCCGTGCAAAGAAGA | Rice synteny |
|  |  |  |  |  | *R:* GGAGACGAGGAGCAAAAGACACAA |  |
| WBE308 | CAPS | 2H | ClaI | 65 | *F:* CTGAGCCTGGGAAACAAAGTCG | Rice synteny |
|  |  |  |  |  | *R:* CAGCGCTGATGCAACAATAGGAT |  |
| Bmac0216a | SSR | 2H |  |  | *F:* GTACTATTCTTTGCTTGGGC | Ramsay et al. (2000) |
|  |  |  |  |  | *R:* ATACACATGTGCAAAACCATA |  |
| Bmag0125a | SSR | 2H |  |  | *F:* AATTAGCGAGAACAAAATCAC | Ramsay et al. (2000) |
|  |  |  |  |  | *R:* AGATAACGATGCACCACC |  |
| GBM1440a | SSR | 2H |  |  | *F:* CTACCGAGCTCCTCCTCCTC | Marcel et al. (2007) |
|  |  |  |  |  | *R:* GGCCTCCTTCTTGTCGTAGA |  |
| scsnp06130a | CAPS | 2H | HinfI | 56 | *F:* GACGTCCCTCGCGTAAATGG | Rostok et al. (2005) |
|  |  |  |  |  | *R:* TTGGCCGGGAACTTATGGTG |  |
| ^a^ The markers which were mapped near but outside the flanked QTL interval  ^b^ The references for CAPS and SCAR markers give the origin of the sequences obtained for marker development. | | | | | | |

| **Table S2** List of molecular markers mapped at *rphq16*. | | | | | | |
| --- | --- | --- | --- | --- | --- | --- |
| Name | Type | Chrom. | Restriction enzyme | Tm  (^o^C) | Primer sequences (5’-3’) | Source^b^ |
| DsT-33 | SCAR | 5H |  | 45 | *F:* GCACACATATTATCATGAAAAAGAGC | BarleyWorld.org |
|  |  |  |  |  | *R:* ACCCCAAATGAGTTTCGATG |  |
| GBS0408 | CAPS | 5H | MseI | 56 | *F:* ATGCCACCCCTTATGAATCCT | Stein et al. 2007 |
|  |  |  |  |  | *R:* TTGCCCGTTGAAAAGTCCA |  |
| GBS0576 | CAPS | 5H | BspLI | 56 | *F:* GTCCGGGCACAAGAACCTC | Stein et al. 2007 |
|  |  |  |  |  | *R:* GGCTGGGCATCATCCTCAA |  |
| GMS002 | SSR | 5H |  |  | *F:* CCGACAACATGCTATGAAGC | Struss and Plieske (1998) |
|  |  |  |  |  | *R:* CTGCAGCAAATACCCATGTG |  |
| MWG2193 | CAPS | 5H | AluI | 56 | *F:* CAAACCCTTGAGGTCAGTTGC | Graner et al. (1991) |
|  |  |  |  |  | *R:* TCAGCTCTAAGATGCAGCACG |  |
| MWG2249 | CAPS | 5H | DdeI | 56 | *F:* GGCATGTGAGGGAAGCAATGG | Graner et al. (1991) |
|  |  |  |  |  | *R:* TGGAGAAGAACGTGTGGGTCG |  |
| scsnp03275 | CAPS | 5H | BglII | 65 | *F:* AACGGCCAGGCTATAACCATCACA | Rostoks et al. (2005) |
|  |  |  |  |  | *R:* CGGCGGCTTCATCAATTTCACTAA |  |
| scsnp03683 | CAPS | 5H | HpyCHY4IV | 56 | *F:* CAACGGCGCCACCTTCTACT | Rostoks et al. (2005) |
|  |  |  |  |  | *R:* CACATACCCCACTGCCATGC |  |
| WBE310 | SCAR | 5H |  | 65 | *F:* GGCGCTTTTGGTTTTCCTGA | Potokina et al. (2008) |
|  |  |  |  |  | *R:* CGGCCTGGTATAATTAAGAGTGTG |  |
| WBE311 | SCAR | 5H |  | 65 | *F:* CCAGAAAGGCGAGGAAGG | Potokina et al. (2008) |
|  |  |  |  |  | *R:* TCGGATTATTGCACACCAGAAAAC |  |
| WBE312 | CAPS | 5H | MseI | 58 | *F:* TGTGCCGTGTTATAATGGGGAATG | Potokina et al. (2008) |
|  |  |  |  |  | *R:* CACAAAATCGGGCCTGCTTATCTT |  |
| WBE313 | CAPS | 5H | MwoI | 58 | *F:* TGCCGAGTCGCCTAACCATA | Potokina et al. (2008) |
|  |  |  |  |  | *R:* TCAACAACTACCTGCCAAATACCA |  |
| WBE314 | CAPS | 5H | SphI | 65 | *F:* CCAGGGAATTACCAGGGAGACA | Potokina et al. (2008) |
|  |  |  |  |  | *R:* TGAAGCCGACAACAAAAACAGG |  |
| WBE315 | CAPS | 5H | HinfI | 65 | *F:* CCCCCTTCGCCGGCTTCTCAACC | Potokina et al. (2008) |
|  |  |  |  |  | *R:* ATTCACAAAGCGCCGGCACACCAG |  |
| WBE317 | CAPS | 5H | AcyI | 65 | *F:* ATCCCAGCCGACAGCATCC | Rice synteny |
|  |  |  |  |  | *R:* GAGAGCAGGCACCCGCATAG |  |
| WBE318 | CAPS | 5H | Hin1II | 65 | *F:* ACGGTGGTGGTGGTGGTCA | Rice synteny |
|  |  |  |  |  | *R:* GCCCGCAGCGTCTCGTAG |  |
| WBE319 | CAPS | 5H | HhaI | 65 | *F:* GATGGGTAGGCTTAAGCAGAAACT | Rice synteny |
|  |  |  |  |  | *R:* AACGCGCCTAACACAAACTCCTAC |  |
| WBE320 | CAPS | 5H | MseI | 58 | *F:* CCCCCGGCTGGTGTGGA | Potokina et al. (2008) |
|  |  |  |  |  | *R:* CAGCTGTGGCGTGATGTATTTGTA |  |
| ABC622^a^ | CAPS | 5H | AluI | 65 | *F:* AGGGAAGGGCTGCAAACTGTA | Rostoks et al. (2005) |
|  |  |  |  |  | *R:* ACCAACTGATCGCTGCCTGTGTAT |  |
| ABG390 ^a^ | CAPS | 5H | AluI | 56 | *F:* TGTTCCCAGCATTTGAACAG | Rostoks et al. (2005) |
|  |  |  |  |  | *R:* CGGCAATCCTAATTTTTGGA |  |
| ABG391 ^a^ | CAPS | 5H | AluI | 56 | *F:* GCAAGTGCACTGCTGTACAA | Rostoks et al. (2005) |
|  |  |  |  |  | *R:* TGTTCTCGTACCATGACTTC |  |
| CMWG650 ^a^ | CAPS | 5H | Hin1II | 56 | *F:* ATGCCTGGGTACAAAAATCAAATG | Stein et al. 2007 |
|  |  |  |  |  | *R:* TCACCCAGCCTACCAAAATAACAG |  |
| GMS001 ^a^ | SSR | 5H |  |  | *F:* CTGACCCTTTGCTTAACATGC | Struss and Plieske (1998) |
|  |  |  |  |  | *R:* TCAGCGTGACAAACAATAAAGG |  |
| scsnp00635 ^a^ | CAPS | 5H | HinfI | 65 | *F:* TGAGCAGCCGTGTCAGCTTC | Rostoks et al. (2005) |
|  |  |  |  |  | *R:* AAACATTGGATTGGGCACGC |  |
| scsnp07825 ^a^ | SCAR | 5H |  | 65 | *F:* GGCGCGGCGGACTGACAAG | Rostoks et al. (2005) |
|  |  |  |  |  | *R:* GTGGTGCTGCGACGAGGAGACG |  |
| scssr03907 ^a^ | SSR | 5H |  |  | *F:* CTCCCATCACACCATCTGTC | Ramsay et al. (2004) |
|  |  |  |  |  | *R:* GACATGGTTCCCTTCTTCTTC |  |
| scssr09041 ^a^ | SSR | 5H |  |  | *F:* CATGTCAGTGGGGTTCTAGC | Ramsay et al. (2004) |
|  |  |  |  |  | *R:* TCTACTTGGACCTGCTGACC |  |
| ^a^ The markers which were mapped near but outside the flanked QTL interval  ^b^ The references for CAPS and SCAR markers give the origin of the sequences obtained for marker development. | | | | | | |

| **Table S3** List of molecular markers mapped at *Rphq15* and *Rphq17*. | | | | | | |
| --- | --- | --- | --- | --- | --- | --- |
| Name | Type | Chrom. | Restriction enzyme | Tm  (^o^C) | Primer sequences (5’-3’) | Source^a^ |
| *Rphq15* | | | | | | |
| Bmag0500 | SSR | 6H |  |  | *F:* GGGAACTTGCTAATGAAGAG | Ramsay et al. (2004) |
|  |  |  |  |  | *R:* AATGTAAGGGAGTGTCCATAG |  |
| GBM1355 | SSR | 6H |  |  | *F:* ATCCGTCGTATTCGCATCTC | Varshney et al. (2006) |
|  |  |  |  |  | *R:* GCTGGTACTGGGAGAAATGG |  |
| GBMS033 | SSR | 6H |  |  | Confidential | Li et al. 2003 |
|  |  |  |  |  |  |  |
| MWG966 | CAPS | 6H | BspLI | 57 | *F:* ATGCGTGCCCTTTGGAACA | Graner et al. (1991) |
|  |  |  |  |  | *R:* TGGCCTGCGATATGGAGACC |  |
| scssr09398 | SSR | 6H |  |  | *F:* AGAGCGCAAGTTACCAAGC | Ramsay et al. (2004) |
|  |  |  |  |  | *R:* GTGCACCTCAGCGAAAGG |  |
|  |  |  |  |  |  |  |
| *Rphq17* | | | | | | |
| Bmac0067 | SSR | 3H |  |  | AACGTACGAGCTCTTTTTCTA | Ramsay et al. (2004) |
|  |  |  |  |  | ATGCCAACTGCTTGTTTAG |  |
| Bmag0136 | SSR | 3H |  |  | GTACGCTTTCAAACCTGG | Ramsay et al. (2004) |
|  |  |  |  |  | GTAGGAGGAAGAATAAGGAGG |  |
| ^a^ The references for CAPS and SCAR markers give the origin of the sequences obtained for marker development. | | | | | | |

**References**

Boyd CN, Horsley R, Kleinhofs A Barley chromosome 2 (2H) bin 10 fusarium head blight resistance QTL: Mapping and development of isolines. In: 2007 Nat Fusarium Head Blight Forum, 2007. Citeseer, p 170.

Graner A, Jahoor A, Schondelmaier J, Siedler H, Pillen K, Fischbeck G, Wenzel G, Herrmann RG (1991) Construction of an RFLP map of barley. Theor Appl Genet 83 (2):250-256.

Hori K, Sato K, Nankaku N, Takeda K (2005) QTL Analysis in Recombinant Chromosome Substitution Lines and Doubled Haploid Lines Derived from a Cross between Hordeum vulgare ssp. vulgare and Hordeum vulgare ssp. spontaneum. Mol Breed 16 (4):295-311.

Li JZ, Sjakste TG, Röder MS, Ganal MW (2003) Development and genetic mapping of 127 new microsatellite markers in barley. Theor Appl Genet 107 (6):1021-1027.

Marcel TC, Varshney RK, Barbieri M, Jafary H, de Kock MJD, Graner A, Niks RE (2007) A high-density consensus map of barley to compare the distribution of QTLs for partial resistance to *Puccinia hordei* and of defence gene homologues. Theor Appl Genet 114 (3):487-500.

Potokina E, Druka A, Luo Z, Wise R, Waugh R, Kearsey M (2008) Gene expression quantitative trait locus analysis of 16 000 barley genes reveals a complex pattern of genome‐wide transcriptional regulation. Plant J 53 (1):90-101.

Ramsay L, Macaulay M, Ivanissevich Sd, MacLean K, Cardle L, Fuller J, Edwards KJ, Tuvesson S, Morgante M, Massari A, Maestri E, Marmiroli N, Sjakste T, Ganal M, Powell W, Waugh R (2000) A simple sequence repeat-based linkage map of barley. Genetics 156 (4):1997-2005

Ramsay L, Russell J, Macaulay M, Booth A, Thomas WTB, Waugh R (2004) Variation shown by molecular markers in barley: Genomic and genetic constraints. Asp Appl Biol 72:147-154

Rostoks N, Mudie S, Cardle L, Russell J, Ramsay L, Booth A, Svensson JT, Wanamaker SI, Walia H, Rodriguez EM, Hedley PE, Liu H, Morris J, Close TJ, Marshall DF, Waugh R (2005) Genome-wide SNP discovery and linkage analysis in barley based on genes responsive to abiotic stress. Mol Genet Genomics 274 (5):515-527.

Stein N, Prasad M, Scholz U, Thiel T, Zhang H, Wolf M, Kota R, Varshney RK, Perovic D, Grosse I, Graner A (2007) A 1,000-loci transcript map of the barley genome: New anchoring points for integrative grass genomics. Theor Appl Genet 114 (5):823-839.

Struss D, Plieske J (1998) The use of microsatellite markers for detection of genetic diversity in barley populations. Theor Appl Genet 97 (1-2):308-315.

Thiel T, Michalek W, Varshney R, Graner A (2003) Exploiting EST databases for the development and characterization of gene-derived SSR-markers in barley (*Hordeum vulgare* L.). Theor Appl Genet 106 (3):411-422.

Varshney RK, Grosse I, Hähnel U, Siefken R, Prasad M, Stein N, Langridge P, Altschmied L, Graner A (2006) Genetic mapping and BAC assignment of EST-derived SSR markers shows non-uniform distribution of genes in the barley genome. Theor Appl Genet 113 (2):239-250.
